# Evaluation of OASIS QSAR Models Using ToxCast™ *in Vitro* Estrogen and Androgen Receptor Binding Data and Application in an Integrated Endocrine Screening Approach

**DOI:** 10.1289/EHP184

**Published:** 2016-05-06

**Authors:** Barun Bhhatarai, Daniel M. Wilson, Paul S. Price, Sue Marty, Amanda K. Parks, Edward Carney

**Affiliations:** Toxicology Environmental Research and Consulting, The Dow Chemical Company, Midland, Michigan, USA

## Abstract

**Background:**

Integrative testing strategies (ITSs) for potential endocrine activity can use tiered in silico and in vitro models. Each component of an ITS should be thoroughly assessed.

**Objectives:**

We used the data from three in vitro ToxCast™ binding assays to assess OASIS, a quantitative structure-activity relationship (QSAR) platform covering both estrogen receptor (ER) and androgen receptor (AR) binding. For stronger binders (described here as AC50 < 1 μM), we also examined the relationship of QSAR predictions of ER or AR binding to the results from 18 ER and 10 AR transactivation assays, 72 ER-binding reference compounds, and the in vivo uterotrophic assay.

**Methods:**

NovaScreen binding assay data for ER (human, bovine, and mouse) and AR (human, chimpanzee, and rat) were used to assess the sensitivity, specificity, concordance, and applicability domain of two OASIS QSAR models. The binding strength relative to the QSAR-predicted binding strength was examined for the ER data. The relationship of QSAR predictions of binding to transactivation- and pathway-based assays, as well as to in vivo uterotrophic responses, was examined.

**Results:**

The QSAR models had both high sensitivity (> 75%) and specificity (> 86%) for ER as well as both high sensitivity (92–100%) and specificity (70–81%) for AR. For compounds within the domains of the ER and AR QSAR models that bound with AC50 < 1 μM, the QSAR models accurately predicted the binding for the parent compounds. The parent compounds were active in all transactivation assays where metabolism was incorporated and, except for those compounds known to require metabolism to manifest activity, all assay platforms where metabolism was not incorporated. Compounds in-domain and predicted to bind by the ER QSAR model that were positive in ToxCast™ ER binding at AC50 < 1 μM were active in the uterotrophic assay.

**Conclusions:**

We used the extensive ToxCast™ HTS binding data set to show that OASIS ER and AR QSAR models had high sensitivity and specificity when compounds were in-domain of the models. Based on this research, we recommend a tiered screening approach wherein a) QSAR is used to identify compounds in-domain of the ER or AR binding models and predicted to bind; b) those compounds are screened in vitro to assess binding potency; and c) the stronger binders (AC50 < 1 μM) are screened in vivo. This scheme prioritizes compounds for integrative testing and risk assessment. Importantly, compounds that are not in-domain, that are predicted either not to bind or to bind weakly, that are not active in in vitro, that require metabolism to manifest activity, or for which in vivo AR testing is in order, need to be assessed differently.

**Citation:**

Bhhatarai B, Wilson DM, Price PS, Marty S, Parks AK, Carney E. 2016. Evaluation of OASIS QSAR models using ToxCast™ in vitro estrogen and androgen receptor binding data and application in an integrated endocrine screening approach. Environ Health Perspect 124:1453–1461; http://dx.doi.org/10.1289/EHP184

## Introduction

The ability to quickly profile and prioritize large numbers of compounds for potential hazards, including endocrine receptor binding activity, improved with the advent of predictive *in vitro* high-throughput screening (HTS) methodologies such as ToxCast™ (Dix et al. 2007; Kavlock et al. 2012). ToxCast™ uses a battery of HTS assays to develop activity signatures across a range of *in vitro* end points and chemistries. ToxCast™ Phase II included approximately 1,800 compounds screened in a subset of assays focusing on potential endocrine activity, which included both biochemical and cell-based measures. Similarly, development of *in silico* predictive QSAR models for toxicity estimations is continuously advancing. Independent QSAR models are available for some of the end points in ToxCast™, such as estrogen receptor (ER) and androgen receptor (AR) binding, which were originally developed using smaller subsets of *in vitro* receptor binding data generated under other platforms. In the present study, we used the extensive ER and AR assay data published for ToxCast™ Phase II to assess the performance and thus to further delineate the validity of the 3D-QSAR model predictions of the ER and AR binding models implemented in OASIS (Mekenyan et al. 2000). The predictions in terms of receptor binding potency are addressed for the ER model. The likelihood of using a tiered approach to flag in-domain compounds predicted as active by QSAR and demonstrated as strong binders in ToxCast™ (AC_50_ or concentration at which activity is 50% of its maximum < 1 μM) to identify compounds that would also be active in respective transactivation and the uterotrophic assay is addressed.

Assessment of the potential endocrine activity of compounds is an area of intense focus worldwide. In the United States, the Endocrine Disruptor Screening Program in the 21st Century (EDSP21) [U.S. Environmental Protection Agency (EPA) 2015] has developed a tiered assessment and prioritization scheme to screen commercial compounds with the potential for consumer exposure. EDSP screening ranges from short-term *in vitro* assays to multigenerational studies in which eleven assays (five *in vitro* and six *in vivo*) are used as a first tier to determine whether compounds interact with three endocrine hormonal pathways—ER, AR and thyroid (U.S. EPA 2015). Compounds that bind to the ER or the AR might influence endocrine signaling by either blocking the binding of endogenous hormones, by activating receptor signaling, or by performing both actions (Katzenellenbogen 1995; Katzenellenbogen et al. 2003). These compounds may also mimic the action of hormones because of their structural similarity and may initiate similar downstream sequelae or alter the concentrations of hormones affecting their synthesis, transport, metabolism and excretion (Katzenellenbogen 1995; Katzenellenbogen et al. 2003). The U.S. EPA ToxCast™ program has made *in vitro* HTS data publicly available for a broad range of cellular and biochemical targets that cover major protein superfamilies, key signaling pathways, and phenotypic end points. In addition, known nuclear receptor (NR) targets, including steroid hormone receptors such as the ER and the AR, are included (Kavlock et al. 2012).

The ER and AR binding assays studied herein were implemented by NovaScreen (NVS), and covered cloned receptors were isolated from three different mammalian species. Other assays that include transactivation- and pathway-based assays were from the Odyssey Thera (OT), Attagene (ATG), ACEA and Tox21 platforms. Uterotrophic assay data were obtained from a curated data set (ER platform) as described recently (Browne et al. 2015). To date, the AC_50_ value has been the most commonly used *in vitro* parameter under ToxCast™ and was used herein (Dix et al. 2007; Kavlock et al. 2012). There are several available *in silico* models for predicting ER and AR activity; these models are summarized elsewhere (Lo Piparo and Worth 2010). Recent manuscripts revealed various approaches such as structure-based (Steinmetz et al. 2015), docking-based (Kolšek et al. 2014), or mathematical models using *in vitro* data (Browne et al. 2015; Judson et al. 2015; McRobb et al. 2014; Zhang et al. 2013) to access ER binding. Here, we evaluated both the qualitative and the quantitative predictivity of the OASIS QSAR model for ER and AR binding and transactivation using the ToxCast™ HTS screening data as the challenge data set. The predictions in OASIS are based on the combination of a toxicodynamic and toxicokinetic model in a single platform, where a mechanistic QSAR model for ER and AR binding affinity is combined with metabolism models [referred to in the QSAR tool as Tissue Metabolism Simulator (TIMES)] to address binding of either the parent compound or its predicted metabolites (Mekenyan et al. 2000). The Common Reactivity Pattern (COREPA) approach (Bradbury et al. 2000; Mekenyan et al. 2000) is implemented in the software, which helps to identify stereo-electronic characteristics associated with a chemical’s biological activity by incorporating dynamic conformational flexibility. For the ER model (trained on human and trout *in vitro* assay data), relative binding affinity (RBA) is predicted relative to 17β-estradiol (100% binding). Nucleophilicity, interatomic distance between electronegative heteroatoms, and electron donor capability of heteroatoms are all important model variables. Similarly, the AR binding affinity model is based on a set of stereoelectronic parameters that provide a maximal measure of pairwise similarity among the conformers of the most active steroidal and nonsteroidal ligands. The standard QSAR model assesses the binding affinity of the parent compounds only, unless the metabolism is switched on or compounds are evaluated with the metabolism simulator before running the models (Shelby et al. 1996). Principal metabolic transformations include oxidative reactions such as aromatic ring hydroxylation and O-dealkylation, which are generated by hepatic cytochrome P450 (CYP) enzymes. In this article, prediction results obtained from the use of ER and AR receptor binding 3D-QSAR models from OASIS-TIMES relative to the ToxCast™ Phase II compounds are presented. The models herein were run without incorporation of the TIMES QSAR metabolism simulation because the ToxCast™ binding assays did not incorporate metabolism. In addition, the outcomes of respective transactivation assays and the uterotrophic assay are presented for the subset of in-domain compounds predicted and shown to be active across all ToxCast™ binding platforms with AC_50_ < 1 μM. The reliability of the QSAR estimate compared with the ToxCast™ assay result and the mechanistic explanation of the QSAR estimation for some compounds are discussed. The utility of ToxCast™ data for the refinement of existing QSAR models is also suggested.

## Methods

### Compounds and Assays

All compounds with data on *in vitro* ER and AR assays were obtained from the ToxCast™ Phase II public release on 5 December 2013 (Dix et al. 2007). These assays span across different species, targets, genes, and so forth (see Table S1). Compounds without defined structures, for example, oils or mixtures such as milbemectin(containing milbemycin A4 and milbemycin A3), were excluded from the evaluation. For compounds with more than one component, salts or acids were removed, and only the unique parent compound was predicted by the model. Because the bioactivity (AC_50_) data of the parent and the salts were different, for comparison purposes, the original compound name and structure were kept the same as that provided by ToxCast™. The total chemical lists, CAS numbers, SMILES codes, corresponding ToxCast™ assay values, potency bins, and calculated RBA values are given in Excel File Table S2a. In addition, uterotrophic response data were obtained from a recent publication (Browne et al. 2015) for 42 compounds that comprised a subset of the ToxCast™ dataset. Seventy-two reference chemicals used in developing an integrated model for validating ToxCast™ ER assays (Judson et al. 2015) were also compared.

### QSAR Modeling

OASIS (v.2.27.13) predictions for receptor-mediated end points of ER and AR were calculated using estrogen binding affinity (v.03) and androgen binding affinity (v.03) 3D-QSAR models (Mekenyan et al. 2000). The ER model was built with 823 compounds in the training set, which contained 650 human ER and 173 trout ER relative binding affinity (RBA) data points (Katzenellenbogen et al. 2003; Serafimova et al. 2007). Similarly, the AR model was built with 202 compounds in the training set with observed RBA based on recombinant rat protein expressed in *Esccerichia coli*, whose ligand-binding domain is considered to be similar to human AR (Fang et al. 2003; Kelce et al. 1994; Mekenyan et al. 1997; Waller et al. 1996). Both ER and AR models were based on cell-free competitive radio-labeled receptor binding *in vitro* assays. The training set compounds were also ranked for RBA for ER and AR. These models were applied in a batch mode to the compounds based on SMILES information published by the U.S. EPA as part of the ToxCast**™** data set (Dix et al. 2007; Kavlock et al. 2012). Each of the modeled end points was run individually, and the results were exported as a tab-delimited text file. The AM1 Hamiltonian method for MOPAC calculation and “Accurate” conformer generation were selected. The models were run using only OASIS without simulation of metabolism by TIMES. The applicability domain of the models was studied based on the default values selected for total domain estimation as defined in the program. The total domain was a combination of structural-, mechanistic-, and parametric-based domains; compounds not satisfying the criteria in any of the sub-domains were out-of-domain in total.

### Performance of QSAR Models

The performances of the ER and AR QSAR models for ToxCast™ compounds for individual ER and AR assays related to the mammalian nuclear receptor (NR) targets were studied based on specificity, sensitivity, and concordance. In addition, the potency of binding predictions for *in vitro* compounds was calculated based on the RBA compared with that of the positive control (estradiol for ER), converted into percentiles, and compared with the respective *in silico* predictions. No potency-based prediction for AR binding was performed because we were unable to obtain AC_50_ values for the positive control (i.e., R1881). The *in silico* and *in vitro* predictions were assigned into four different bins for ER: RBA > 10% of positive control (denoted as high), 0.1% ≤ 10% of positive control (medium), 0.001% ≤ 0.1% of positive control (low) and from 0% < 0.001% (very low). If the OASIS prediction for a chemical was uncertain and was assigned into two bins, the most conservative prediction bin (i.e., the highest predicted activity) was chosen. The impact on the probability of being positive *a priori* in the assay was also calculated using Bayesian statistics. These calculations were performed for both the in-domain compounds and the total compounds.

### Heat Maps for ER and AR Assays

Heat maps for ER- and AR-related assays were generated using TIBCO Spotfire (http://spotfire.tibco.com/) to index the representative performance of individual assays for the subset of compounds that showed consistent agreement of ER or AR binding at AC_50_ < 1 μM (strong binders). Colors were coded to indicate inactive and active with different ranges of *in vitro* potency.

## Results

### ER and AR QSAR Model Performance for the Binding Assays

The performance of the ER and AR QSAR models for predicting the binding activity of in-domain compounds (by the respective QSAR model) versus the ToxCast™ *in vitro* binding test results are summarized in Table 1. The top half of each table shows the results for ER compounds, and the lower half of each table shows the results for the AR compounds. Similar tables for all compounds are given in Table S2b.

**Table 1:** Summary performance of quantitative structure-activity relationship model predictions for ToxCast™ Phase II compounds against individual mammalian *in vitro* assays: 458 in-domain compounds for estrogen receptor (ER) binding model v.03 (top) and 213 (134 for chimpanzee) in-domain compounds for androgen receptor (AR) binding model v.03 (bottom).

ER binding	Human			Bovine			Mouse		
	Positive	Negative	Total	Positive	Negative	Total	Positive	Negative	Total
Positive	44	13	57	31	3	34	31	10	41
Negative	43	358	401	56	368	424	56	361	417
Total	87	371	458	87	371	458	87	371	458
Sensitivity (%)	44/57 = 77.2			31/34 = 91.2			31/41 = 75.6		
Specificity (%)	358/401 = 89.3			368/424 = 86.8			361/417 = 86.6		
Concordance (%)	(44 + 358)/458 = 87.8			(31 + 368)/458 = 87.1			(31 + 361)/458 = 85.6		
Coverage	[(44 + 358)/1,845] × 100 = 21.8%			[(31 + 368)/1,845] × 100 = 21.6%			[(31 + 361)/1,845] × 100 = 21.2%		

AR binding	Human			Chimpanzee			Rat		
	Positive	Negative	Total	Positive	Negative	Total	Positive	Negative	Total
Positive	36	2	38	25	0	25	23	2	25
Negative	33	142	175	32	77	109	46	142	188
Total	69	144	213	57	77	134	69	144	213
Sensitivity (%)	36/38 = 94.7			25/25 = 100			23/25 = 92		
Specificity (%)	142/175 = 81.1			77/109 = 70.6			142/188 = 75.5		
Concordance (%)	(36 + 142)/213 = 83.6			(25 + 77)/134 = 76.1			(23 + 142)/213 = 77.5		
Coverage	[(36 + 142)/1,758] × 100 = 10.1%			[(25 + 77)/958] × 100 = 10.6%			[(23 + 142)/1,758] × 100 = 9.38%		

For the ER QSAR model, 458 (24.8%) of 1,845 compounds were in-domain, and 1,365 (74%) were out-of-domain. The model assigned “No domain” for 17 (0.9%) compounds, and no information was provided for 5 (0.3%) compounds. The ER QSAR model had both high sensitivity (> 75%) and specificity (> 86%) for in-domain compounds. ER QSAR predictions had low sensitivity (< 56%) but high specificity (> 95%) when the model was applied without distinction of domain boundaries. Of the 458 in-domain compounds, 87 (19.0%) were predicted to be active, and 371 (81.0%) were predicted to be inactive. For in-domain compounds, the overall concordance decreased by ~5%, and sensitivity increased by 36–38% compared with predictions for the total compound data set.

For the AR QSAR model, 213 (12.1%) of 1,758 compounds were in-domain, and 1,516 (86.2%) were out-of-domain. The model assigned “No domain” for 17 (0.9%) compounds, and no information was provided for 12 (0.7%) compounds. The AR QSAR model had both high sensitivity (92–100%) and specificity (70–81%) for in-domain compounds. AR QSAR model predictions had low sensitivity (< 41%) but high specificity (84–89%) when the model was applied without distinction of domain boundaries. Of the 213 in-domain compounds, 69 (32.4%) were predicted to be active, and 144 (67.6%) were predicted to be inactive. For in-domain compounds, the overall concordance decreased by ~10%, and sensitivity increased by 53–64% compared with predictions for the total compound dataset.

### Consideration of Stronger ER and AR Binders

**Figure 1:**
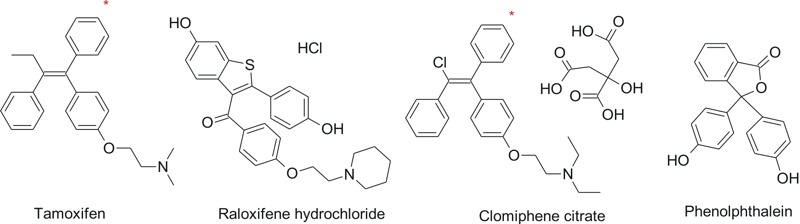
Tamoxifen, clomiphene (which are triphenylethylenes), and raloxifene (which is a benzothiophene) are common estrogen receptor binders used in clinical practice for treatment of breast cancer, induction of ovulation in subfertile women, and prevention of postmenopausal osteoporosis, respectively. Phenolphthalein, used in nonprescription laxative preparations, also has a weak estrogenic action (Ravdin et al. 1987). The site of 4-hydroxylation for tamoxifen and clomiphene is indicated with an asterisk.

For both the ER and AR QSAR models, when the HTS results for all three mammalian nuclear receptor binding assays were restricted to those showing consistent agreement of binding at AC_50_ < 1 μM, the QSAR models accurately predicted binding for the parents or known hydroxylated metabolites 100% of the time. There were 20 compounds for ER (Table 2) and 11 for AR (Table 3) for which the HTS results for all three binding assays showed consistent agreement for binding at AC_50_ < 1 μM. For the ER QSAR model, 3 compounds (clomiphene, tamoxifen and tamoxifen citrate; Figure 1) were out of the domain of the model, but their known mammalian 4-hydroxyphenyl metabolites satisfied the structural domain boundary requirements. Clearly, the domain of ER model is constrained to phenols, which is consistent with the fact that 4-hydroxy-tamoxifen was in-domain and ~10 times more potent a binder than either tamoxifen or its citrate salt, which were considered out-of-domain by the model. Thus, for the ER model, considering not only the parent compounds but their hydroxylated metabolites was necessary to obtain the 100% prediction. Both parent compounds and hydroxylated metabolites bound strongly (AC_50_ < 1 μM) in the ToxCast™ assays. Two other compounds (raloxifene hydrochloride and phenolphthalein) were also strong binders *in vitro* but were predicted to be out-of-domain by the ER QSAR model. Thus, such data could be used to improve the QSAR model. For the AR QSAR model, all 11 compounds were predicted to be active and in-domain 100% of the time except for mifepristone, which was not predicted and was out-of-domain.

**Table 2:** Twenty compounds with estrogen receptor (ER) binding at AC_50_ < 1 μM for all three mammalian nuclear receptor binding assays. The *in silico* prediction results including the total domain information and the *in vitro* assay data are given.

Compound name	Predicted result	Total domain	NVS_NR_bER	NVS_NR_hER	NVS_NR_mERa
Clomiphene citrate^*a*^	Not active	Out of Domain	0.0317	0.00975	0.187
17α-Estradiol	Active	In domain	0.000493	0.0000595	0.0229
17β-Estradiol	Active	In domain	0.000174	0.0229	0.00164
4-Hydroxytamoxifen	Active	In domain	0.00186	0.0025	0.0723
Diethylstilbestrol	Active	In domain	0.0229	0.0229	0.00632
17α-Ethinylestradiol	Active	In domain	0.000245	0.0000541	0.00185
Bisphenol A	Active	In domain	0.389	0.131	0.15
Bisphenol AF	Active	In domain	0.096	0.0449	0.0242
Bisphenol B	Active	In domain	0.149	0.0291	0.022
2,2’,4,4’-Tetrahydroxybenzophenone	Active	In domain	0.268	0.0534	0.176
Daidzein	Active	In domain	0.481	0.116	0.173
Estriol	Active	In domain	0.00763	0.0229	0.0421
Estrone	Active	In domain	0.104	0.000795	0.00763
*meso*-Hexestrol	Active	In domain	0.000277	0.0229	0.0229
1,1,1-Trichloro-2,2-bis(4-hydroxyphenyl)ethane	Active	In domain	0.0176	0.0392	0.00985
Genistein	Active	In domain	0.0983	0.0167	0.0901
Raloxifene hydrochloride^*a*^	Not active	Out of Domain	0.00763	0.0000476	0.0253
Phenolphthalein	Active	Out of Domain	0.658	0.887	0.228
Tamoxifen	Not active	Out of Domain	0.0834	0.0349	0.133
Tamoxifen citrate^*a*^	Not active	Out of Domain	0.106	0.0246	0.223
^***a***^The salt or the acid component is removed for quantitative structure-activity relationship (QSAR) modeling.					

**Table 3:** Eleven compounds with androgen receptor (AR) binding at AC_50_ < 1 μM for all three mammalian nuclear receptor binding assays. The *in silico* prediction results including the total domain information and the *in vitro* assay data are given.

Compound name	Predicted result	Total domain	NVS_NR_cAR	NVS_NR_hAR	NVS_NR_rAR
17α-Estradiol	Active	In domain	0.024	0.0057	0.242
17β-Estradiol	Active	In domain	0.0167	0.00293	0.12
17β-Trenbolone	Active	In domain	0.00744	0.000201	0.0179
17-Methyltestosterone	Active	In domain	0.00614	0.00144	0.0802
5α-Dihydrotestosterone	Active	In domain	0.00763	0.000566	0.022
Cyproterone acetate^*a*^	Active	In domain (belongs to training set)	0.0326	0.00763	0.258
Mifepristone	Cannot predict	Out of Domain	0.0388	0.0282	0.0621
Norethindrone	Active	In domain	0.00396	0.000505	0.147
Norgestrel	Active	In domain	0.00538	0.00131	0.093
Progesterone	Active	In domain	0.163	0.00763	0.414
Spironolactone	Active	In domain	0.0136	0.00303	0.254
^***a***^The salt or the acid component is removed for quantitative structure-activity relationship (QSAR) modeling.					

### Heat Maps for ER and AR Assays

**Figure 2:**
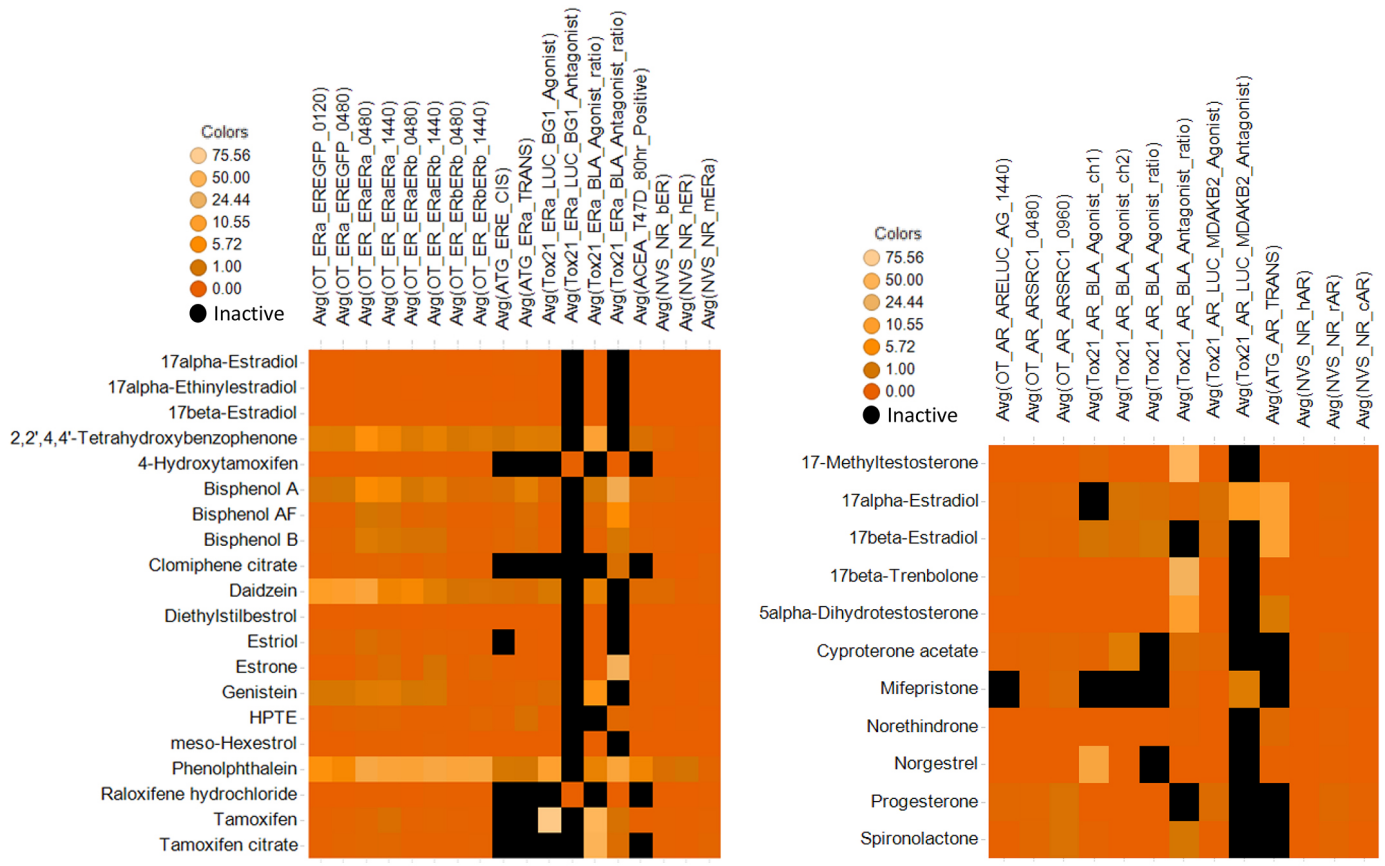
Heat maps of 18 estrogen receptor (left) and 11 androgen receptor (right) compounds with AC_50_ < 1 μM (most active) for ToxCast™ assays using TIBCO Spotfire (http://spotfire.tibco.com/). Color codes indicate least to most active compounds with the increasing gradient of brown color: darker shades indicate more potent activity (lower AC_50_), and black represents inactive compounds.

Heat maps were generated for the subset of compounds that showed consistent agreement of ER or AR binding at ToxCast™ AC_50_ < 1 μM and where the QSAR models predicted in-domain binding for the parents or known hydroxylated metabolites 100% of the time. The heat maps (Figure 2) show the distribution of ToxCast™ *in vitro* activity for each assay and are color-coded by potency. This subset of compounds was active in all ER and AR transactivation assays where metabolism was incorporated with addition of an exogenous S9 fraction (OT platform). For those compounds in this subset known to require metabolism to manifest activity, the transactivation response appears to be less promiscuous than binding because of the mixed nature of the response in these assays, which may reflect some degree of constitutive metabolism depending on cell type.

For ER, the heat map (Figure 2) shows the 18 assays that were selected by the U.S. EPA (Judson et al. 2015) to derive an integrated model for ER pathway perturbation and were also included as component assays of the EDSP21 Dashboard (U.S. EPA 2015). Figure 2 shows that the ER compounds were inactive in the antagonist assays, Era_BLA_Antagonist and Era_LUC_BG1_Antagonist, except for 4-hydroxytamoxifen and raloxifene, which are known selective ER modulators (SERMs). Four of the compounds known to undergo further metabolism in relation to ER activity (tamoxifen and its citrate salt, clomiphene citrate and raloxifene hydrochloride) were inactive in half of the ER assays that did not include metabolism but were active in those that did. Notably, 4-OH tamoxifen was inactive in the assays that did not include metabolism and was active in those that did; this was an interesting observation in view of its known ER binding.

**Figure 3:**
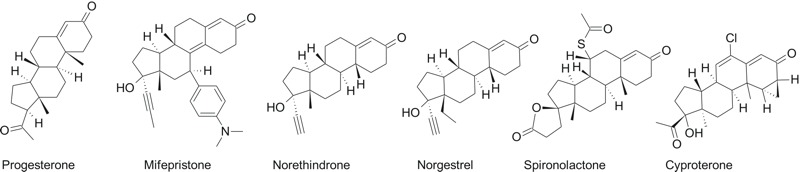
Progesterone and its synthetic analogs which are progesterone receptor binders and in higher doses can bind the androgen receptor. They are used in clinical practice to induce abortion, treat premenstrual syndrome and pain, as hormonal contraceptives, and to reduce elevated or unwanted androgen activity in the body, respectively.

Similarly, for AR, all selected compounds were active in all assays except for two, Tox21_AR_LUC_MDAKB2_Antagonist and Tox21_AR_BLA_Antagonist_ratio, which was expected because the compounds were agonists. The ATG_AR_TRANS assay exhibited differential activation to some known progesterone receptor agonists (such as spironolactone, mifepristone, cyproterone) but not to all (norethindrone and norgestrel) (Figure 3). Our findings highlight the ability to use QSAR models to predict those compounds that were uniformly active in all respective ToxCast™ assay platforms with the caveat of needing to account for some metabolic activation.

### ER QSAR Model Performance for the Uterotrophic Assay

We further examined the ability of the ER QSAR model to predict the activity of *in vivo* responses in a curated uterotrophic data set (Browne et al. 2015). The sensitivity of the QSAR predictions averaged > 80% for those compounds in-domain of the model (see Table S3a). When the compounds in this group were restricted to the subset of strong binders (AC_50_ < 1 μM), the results showed that a QSAR prediction of receptor binding for in-domain compounds identified active compounds of other *in vitro* ER assays and those in the *in vivo* uterotrophic assay. Notably, all of the compounds in this restricted group were identified as actives used in the training set of the ER QSAR model (see Table S3b).

### Application of ER QSAR to 72 Reference Compounds

The selected ToxCast™ ER-related 18 assays and 72 reference compounds were studied in a recent publication (Judson et al. 2015). These 72 compounds were a subset of ToxCast™ compounds and were chosen to validate the ER assays. We assessed these compounds and assays using the OASIS QSAR model in the present study. There were 27 reference compounds with AC_50_ < 1 μM, of which all except phenolphthalein were strong binders (i.e., AC_50_ < 1 μM) in the ER ToxCast™ assay list. When the strong binders observed in all three ER-binding ToxCast™ assays were compared with the 72 reference compounds (Judson et al. 2015), two strong binders in the ER ToxCast™ assay list (daidzein and 2,2´,4,4´-tetrahydroxybenzophenone) had median AC_50_ values > 1 μM, whereas 10 of 70 reference compounds with AC_50_ < 1 μM were not strong binders in the ER ToxCast™ assay list. Compared with the 42 uterotrophic compounds (Browne et al. 2015), there were 25 compounds evaluated in the reference compound list.

### *In Vitro* Binding Potency Estimation

The predicted potency of *in vitro* ER binding from the QSAR model was compared with the RBA of 17β-estradiol, the positive control used in deriving the RBA of the ER QSAR model. The RBAs for the investigated compounds were calculated relative to the AC_50_ value of 17β-estradiol and converted into percentiles. Comparison of RBA levels for *in silico* versus *in vitro* was a poorer match for the high and medium binding levels of the human receptor than the other two species. This phenomenon was observed for both in-domain and total compounds because most of the compounds that were not predicted were in the “None” category (Table 4). For AR binding, the potency-based prediction was not performed because we were unable to obtain AC_50_ values for R1881, the positive control used to derive the RBA of the AR QSAR model. The AC_50_ values were not available in the raw data files for R1881. Correspondence with a research scientist at the U.S. EPA (M. Martin, written communication, October 2014) suggested that the R1881 was used as a positive control at a couple of concentrations only, and the full concentration response was not obtained. In addition, the AC_50_ was not calculated based on the potency performance of the positive control (e.g., relative binding); instead, the positive control wells were normalized to derive response values (efficacy) that were modeled across the tested concentration range for the entire ToxCast™ chemical library. The Office of Prevention, Pesticides and Toxic Substances (OPPTS) EDSP test guideline for conducting the AR binding assay using rat prostate cytosol suggests blocking the potential interaction of the ligand with progesterone receptors as part of the assay procedure (U.S. EPA 2009). Based on personal communication with a research scientist at the U.S. EPA (M. Martin, written communication, October 2014), the procedures of the NVS assay did not address such matters.

**Table 4:** Summary performance of quantitative structure-activity relationship model potency predictions for in-domain ToxCast™ Phase II compounds against individual mammalian *in vitro *assays for estrogen receptor binding model v.03.

In domain *in vitro*	Bovine						Human						Mouse					
	*In silico*					Total	*In silico*					Total	*In silico*					Total
	Hi	Med	Low	VLow	None		Hi	Med	Low	VLow	None		Hi	Med	Low	VLow	None	
Hi	**3**	1	0	0	0	4	**8**	7	***7***	0	2	24	**3**	2	0	0	0	5
Med	5	**6**	***1*****	0	0	12	1	**6**	***11***	***4***	7	29	4	**3**	***8***	0	1	16
Low	0	1	**5**	0	1	7	0	0	**0**	0	4	4	0	4	**3**	1	10	18
VLow	0	***4***	3	**2**	2	11	0	0	0	**0**	0	0	0	0	0	**0**	2	2
Total	8	12	9	2	3	34	9	13	18	4	13	57	7	9	11	1	13	41
Abbreviations: Hi, high; Med, medium; VLow, very low. Data are bolded to show agreement and italicized and bolded to show disagreement. Potency bins are described in “Performance of QSAR Models” under “Methods.”																		

### Probability of Positive and Negative Predictions

The value of the QSAR findings for improving the identification of ER and AR binding was determined using Bayesian analyses. The fractions of the tested compounds in ToxCast™ Phase II were used as the “prior” and the “posterior” probabilities of being positive or negative in the assay for all compounds as well as for those in the domain of the respective model. The probabilities were determined for each of the three ER and AR binding assays (Table 5). For ER, the value of the QSAR was greatest for the in-domain compounds, where a positive QSAR prediction indicated a 51% chance of being positive in the human ER assay, and a negative finding indicated a 96% chance of being negative in the human ER assay. For AR, the value of the QSAR was greatest for the in-domain compounds, where a positive QSAR prediction indicated a 52% chance of being positive in the human AR assay, and a negative finding indicated a > 99% chance of being negative in the human AR assay.

**Table 5:** Value of quantitative structure-activity relationship findings in improving the prediction of estrogen receptor binding (left) and androgen receptor binding (right).

Findings	Estrogen receptor						Androgen receptor					
	ToxCast™ (all) cells			ToxCast™ (in-domain) cells			ToxCast™ (all) cells			ToxCast™ (in-domain) cells		
	Human	Bovine	Mouse	Human	Bovine	Mouse	Human	Chimp	Rat	Human	Chimp	Rat
Probability of positive in ToxCast™ data set (%)	6.7	3.5	5.6	12	7.4	9	7	10	6	18	19	12
Probability of positive if QSAR is positive (%)	41	28	30	51	36	36	25	26	17	52	44	33
Probability of negative in ToxCast™ data set (%)	93.3	96.5	94.4	88	92.6	91	93	80	94	82	81	88
Probability of negative if QSAR is negative (%)	96	98	96	96	99	97	95	92	95	99	100	99
Abbreviations: Chimp, chimpanzee; QSAR, quantitative structure-activity relationship.												

Assessment of the QSAR models and the assays used to derive them is also crucial to understand the internal predictivity of the model and for comparison with the ToxCast™ assays. The following sections deal with the predictive ability of the models for the ToxCast™ compounds, present a comparison of the assays used to derive the QSAR models with the ToxCast™ assays, and address the internal predictivity of the QSAR models.

### Estrogen Receptor QSAR Prediction for ToxCast™ Compounds

Of the 1,851 ToxCast™ compounds with *in vitro* ER assay AC_50_ data for all mammalian NR targets, 6 compounds were not predicted by the ER QSAR model; of these, 5 were not active in ≥ 2 assays, and one (fulvestrant) was active in all three ER assays. Of the remaining 1,845 compounds that were predicted, 1,680 (91%) were not active in any of the three ToxCast™ *in vitro* assays, but 74 were predicted to be active by the *in silico* model. Of these 74 compounds, 41 were in the training set of the model with existing experimental data; among these, 9 were not active, and 32 compounds were active with a different range of RBA activity (0% < RBA ≤ 10%). Similarly, of 1,845 compounds, 45 (2.5%) were active in all three ToxCast™ assays; of these 45, 28 were predicted to be active (20 in the training set of the ER model), and 17 were predicted to be not active (none in training set) by the *in silico* model. The remaining 120 compounds were active in some assays and inactive in others.

### Androgen Receptor (AR)QSAR Prediction for ToxCast™ Compounds

Of 1,851 ToxCast™ Phase II compounds for AR, 93 were not predicted by the AR QSAR model (undefined in the range 0.001% < RBA < 0.1% and with missing parameters); of these 93, 66 were not active, 5 were active in all three AR *in vitro* assays, and the remaining 22 had different assay results. Of the remaining 1,758 compounds that were predicted, 800 (45.5%) did not have a value for the chimpanzee AR binding assay (NVS_NR_cAR), and excluding those, 775 (44.1%) were not active in any of the three ToxCast™ *in vitro* assays, although 74 of these were predicted active by the *in silico* model. Of these 74 compounds, 10 were in the training set of the model with experimental *in vivo* data, where 1 was not active, and 9 were active with a different range of RBA activity (0.001% ≤ RBA ≤ 0.1%). Similarly, 39 (2.2%) compounds were active in all three ToxCast™ assays; of these, 20 were predicted active, 14 were predicted not active, and 5 were not predicted by the *in silico* model.

### Comparison of the ER Assay Used for the QSAR Model versus ToxCast™ ER Binding Assays

**Figure 4:**
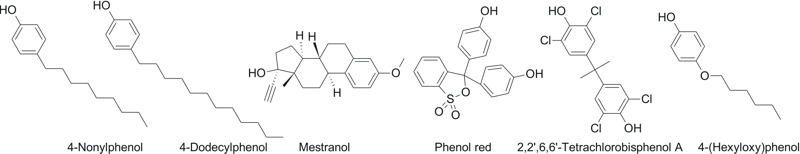
Representative compounds for which *in vivo* and *in vitro* assay results are not concordant. These compounds were present in the training set of the estrogen receptor quantitative structure-activity relationship model, and the data were compiled from *in vivo* studies (Serafimova et al. 2007). The first three compounds were active *in vivo* but inactive *in vitro*, whereas the last three were active *in vitro* but inactive *in vivo*.

Restricting the analysis to compounds with relatively high *in vitro* activity, that is, compounds with RBA > 0.1% that were in the ER QSAR training set (17 compounds), we found 14 to be active in all 3 ToxCast™ *in vitro* assays of mouse, bovine, and human. Of these, 4-nonylphenol (linear) was inactive in all 3 ToxCast™ *in vitro* binding assays (but active in 10 of 11 of the remaining agonist-mode assays), 4-dodecylphenol was inactive in 2 of 3 assays (bovine and mouse), and mestranol was inactive in only 1 (mouse) of 3 ToxCast™ assays (see Figure 4). Similarly, for inactive compounds present in the ER QSAR model (107 compounds), 1, 9 and 7 were active in the bovine, human, and mouse ToxCast™ assays, respectively. Phenolphthalein was active in all three ToxCast™ assays, whereas phenol red, 2,2´,6,6´-tetrachlorobisphenol A, and 4-(hexyloxy)phenol were active in 2 (human and mouse) of 3 assays. Some compounds such as phthalates were considered active in the training set for the ER QSAR model but were inactive in the ToxCast™ *in vitro* assay (see Table S3c). This outcome highlights the contradictory assay results for the same compounds between different *in vitro* assay platforms and ToxCast™.

### Comparison of the AR Assay Used for the QSAR Model versus ToxCast™ AR Binding Assays

Restricting the analysis to compounds with relatively high activity (RBA > 0.1%) that were in the AR QSAR training set (46 compounds), only 2 (cyproterone acetate and hydroxyflutamide) were active in all three ToxCast™ *in vitro* assays of rat, chimpanzee, and human. Similarly, all 30 compounds that were inactive in the training set of the model were inactive or not predicted (NA) in all three ToxCast™ *in vitro* assays.

### QSAR Model Internal Predictivity of Training Set Compounds

In some cases, some of the training set compounds that were used to derive the model were either positive in the *in vitro* ER binding assay but the QSAR model predicted no binding activity (see Table S4a), or were negative *in vitro* but predicted to be positive by the QSAR model (see Table S4b). Similarly, some compounds were either positive in the AR assay and belonged to the training set of the model but were predicted to be negative by QSAR model (see Table S5a), or they were negative *in vitro* but predicted to be positive by the QSAR model (see Table S5b). Out of 190 compounds with relatively high RBA activity in the ER QSAR model, 31 were predicted to be out of the total applicability domain of the model. Similarly, out of 76 compounds with relatively high RBA activity in the AR QSAR model, 19 were predicted to be out of the total applicability domain of the model. The full list of compounds with their names, SMILES codes, *in vitro* assay values, and *in silico* predictions is given in Excel File Table S2a.

## Discussion

The OASIS 3D QSAR models that were used to estimate ToxCast™ *in vitro* data do not have 100% predictive capability. There are active compounds that belonged to the training set of the model and were in-domain, but the QSAR model predicted no binding activity. Moreover, some of the compounds were predicted to be opposite in activity by the ER or AR QSAR models. This lack of predictivity and these opposing predictions could be caused by an undefined RBA activity or by some missing parameters needed for the prediction, thus reducing the prediction performance of the models when using them to further evaluate novel compounds. We also noticed contradictory assay results for the same compounds found in the *in silico* model and ToxCast™ *in vitro* data. Because the *in vitro* assay data platform used to develop the OASIS-QSAR model is different than that of the ToxCast™ assay, we expected to find subtle differences in assay activities and predictions, particularly for weak binders. The differences in assay activities were found more often in the ER platform than in the AR platform for the same compounds.

The sensitivity and specificity of the *in silico* models for binding assays were > 80% on average for the in-domain compounds. This result reveals that a robust and predictive QSAR model developed on multiple assay and species (*in vitro* human and trout data) platforms can be used to predict other *in vitro* and/or *in vivo* data generated in different laboratories, species, or assay platforms. When the evaluation was restricted to include only strong binders (AC_50_ < 1 μM), the prediction of binding for the parents or for known hydroxylated metabolites improved the sensitivity of the predictions to 100% for both the ER and AR models. When the activities of this restricted set were further examined for the other *in vitro* ToxCast™ assays within the ER or AR platforms or for curated uterotrophic data (ER platform), the results showed that a QSAR prediction of receptor binding for in-domain compounds flagged compounds that were always active in the other *in vitro* or *in vivo* screening assays. This finding suggests a tiered screening approach wherein QSAR is first used to identify compounds that are in-domain in the ER or AR binding models and are predicted to bind, which are then screened *in vitro* to assess binding potency, with the strong binders (AC_50_ < 1 μM) screened *in vivo*. It is important to emphasize that this approach would only identify the subset of compounds that are in-domain in the QSAR, flagged as potential binders, and then shown to bind with strong affinities across three independent platforms. This approach would not apply to compounds that are not in domain (majority of compounds), that are predicted not to bind, that require metabolism to manifest activity, that were not active in *in vitro* binding assays, or for the AR platform, where the relationship of the QSAR or *in vitro* assays to *in vivo* data was not studied. Because the binding assays do not accommodate metabolism, consideration should be given to simulating it within the initial QSAR model analysis. For compounds predicted by QSAR to only bind after being metabolized, any negative binders would need to be followed up by other screening approaches that include metabolism.

The ToxCast™ assays can be a robust source of data to improve the existing model predictions, to derive novel *in silico* models with improved predictivity, or to refine the existing QSAR model. For example, in the case of phthalates, which are known to be ER inactive (Moore 2000) (see uterotrophic assay data as well as ToxCast™ *in vitro* binding data), the data used in the derivation of the TIMES ER QSAR model considered them to be active compounds. Phthalates undergo ester hydrolysis *in vivo*, which might explain the discrepancy between the *in vitro* and *in vivo* assay results. Interestingly, compounds such as butyl benzyl phthalate (BBP) (Picard et al. 2001) are active in almost all ToxCast™ ER assays except the NVS ER binding assays. Further analyses are needed to address quantitative prediction of low or medium levels of ER and AR binding using *in silico* approaches. The differences in ER and AR binding activity across species (human, bovine, rat, mouse, chimpanzee, etc.) need to be explored further.

## Conclusions

We assessed the OASIS 3D-QSAR models for predicting ER and AR binding by using *in vitro* HTS binding data from > 1,800 ToxCast™ Phase II compounds generated by NVS. Our analysis indicated that for ER, the QSAR predictions of the three NVS assay platforms’ results had low sensitivity (< 56%) but high specificity (95%) and concordance (> 91%) when all compounds in the data set were analyzed. For the in-domain compounds, the ER QSAR model had high sensitivity (> 75%) and high specificity (> 86%), and overall concordance decreased by ~5%. When HTS results were restricted to a subset of compounds within the domain of the ER QSAR model and with consistent agreement of ER binding at AC_50_ < 1 μM for the three binding assays, the ER QSAR model predicted binding for the parent compounds or known hydroxylated metabolites 100% of the time. Similarly, for AR, QSAR predictions of the three assay platform results had low sensitivity (< 41%) but high specificity (84–89%) and concordance (> 83%) when all compounds in the data set were analyzed. For the in-domain compounds, the AR QSAR model had high sensitivity (92–100%), specificity of 70–81%, and overall concordance decreased by ~10%. Similarly, when HTS results were restricted to a subset of compounds in-domain of the AR QSAR model and with consistent agreement of AR binding at AC_50_ < 1 μM for the three binding assays, the QSAR model accurately predicted binding for the parent compounds 100% of the time.

The potency of binding prediction for *in vitro* compounds was estimated only for the ER model, where *in silico* versus *in vitro* comparison was found to be a poorer match for high and medium RBA levels for the *in vitro* human receptor than receptors for the other two species.

Heat maps showed that the subset of ToxCast™ compounds that were in-domain of the ER or AR QSAR models and predicted to be active were active across all ER and AR binding platforms (AC_50_ < 1 μM). This subset of compounds was also active in the respective transactivation assays, where metabolism was incorporated with the addition of exogenous S9 fraction (OT platform). For the compounds in this subset known to require metabolism to manifest activity, the transactivation response appears to be less promiscuous than binding because of the mixed nature of the response in these assays, which may reflect some degree of constitutive metabolism depending on the cell type. This same subset of ER-active compounds was active in the uterotrophic assay *in vivo*. Based on this research, a tiered screening approach could be implemented wherein *a*) QSAR is used to identify compounds in-domain of the ER or AR binding models and predicted to bind; *b*) those compounds are screened *in vitro* to assess binding potency; and *c*) the stronger binders (AC_50_ < 1 μM) are screened *in vivo*. It is important to emphasize that this approach would only identify the subset of compounds that were in-domain of the QSAR, flagged as potential binders, and then shown to bind with strong affinities across three independent platforms. This approach would not apply to compounds that are not in-domain, that are predicted not to bind, that require metabolism to manifest activity, that are not active in *in vitro* binding assays, or for the AR platform, where the relationship of the QSAR or *in vitro* assays to *in vivo* data has not been studied. In such scenarios, mathematical models using a battery of *in vitro* assays could be useful.

## Supplemental Material

(268 KB) PDF

(381 KB) ZIP
