# Supplementary material for: Evaluation of OASIS QSAR Models Using ToxCast™ in Vitro Estrogen and Androgen Receptor Binding Data and Application in an Integrated Endocrine Screening Approach
Source: Environ Health Perspect. 2016 May 6;124(9):1453–61. doi: 10.1289/EHP184 (PMC5010395; doi:10.1289/EHP184)
Supplement: (268 KB) PDF [file EHP184.s001.acco.pdf]

Environ Health Perspect
DOI: 10.1289/EHP184

Note to readers with disabilities: *EHP* strives to ensure that all journal content is accessible to all readers. However, some figures and Supplemental Material published in *EHP* articles may not conform to 508 standards due to the complexity of the information being presented. If you need assistance accessing journal content, please contact ehp508@niehs.nih.gov. Our staff will work with you to assess and meet your accessibility needs within 3 working days.

Supplemental Material

Evaluation of OASIS QSAR Models Using ToxCast *in Vitro* Estrogen and Androgen Receptor Binding Data and Application in an Integrated Endocrine Screening Approach

Barun Bhhatarai, Daniel M. Wilson, Paul S. Price, Sue Marty, Amanda K. Parks, and Edward Carney

Table of Contents

Table 1. Details for the ToxCast ER and AR binding and transactivation assays selected during 3D-QSAR based prediction study

Excel File, Table 2a. See "Additional Files" below

Table 2b. Summary performance of QSAR model predictions for **all** ToxCast II compounds against individual mammalian *in vitro* assays for Estrogen Receptor (ER) binding model v.03 (top) and Androgen Receptor (AR) binding model v.03 (bottom).

Table 3a. Summary performance of QSAR model predictions for all 42 and 36 in-domain compounds with uterotrophic bioactivity

Table 3b. Eleven compounds that have ER binding at $AC_{50} < 1$ μM for all the three mammalian nuclear receptor binding assays were also active in the uterotrophic assay. They also belong to the training set data used to derive the ER QSAR model. The *in silico* prediction results including the total domain information as well as *in vitro* assay data are given

Table 3c. Four compounds (3 Phthalates and 1 Kaempferol) belonging to the training set were considered active in the ER *in vitro* assay used to derive the model but where inactive in uterotrophic bioactivity

Table 4a. Sixteen compounds that were active experimentally and belonged to the training set but were predicted not active for ER binding

Table 4b. Fifteen Compounds that were not active experimentally and belonged to the training set but were predicted active for ER binding

Table 5a. Twelve compounds that were active experimentally and belonged to the training set but were predicted not active for AR binding

Table 5b. Two compounds that were not active experimentally and belonged to the training set but were predicted active for AR binding

Additional Files

Supplemental Code and Data Zip File

Supplemental Code and Data Zip File Index

Excel File, Table 2a. The total chemical lists, CAS numbers, SMILES codes, corresponding ToxCast assay values, potency bins, and calculated RBA values

Assay	Assay Description	Species	Gene ID	Gene Symbol	Assay category	Assay technology	Assay target source	Assay target source type
NVS_NR_hER	Human ER 3H-Estradiol	human	2099	ESR1	Competitive Binding	Radioactivity	Pimary Cell	Breast cancer cells
NVS_NR_bER	Bovine ER 3H-Estradiol	bovine	407238	ESR1		Radioactivity	Tissue	Uterus membrane
NVS_NR_mERa	Mouse ERa 3H-Estradiol	mouse	13982	Esr1		NA	Recombinant	Ligand binding domain
NVS_NR_hAR	Human AR 3H-Methyltrienolone	human	367	AR		Radioactivity	Cell line	LnCAP
NVS_NR_rAR	Rat AR 3H-Methyltrienolone	rat	24208	Ar		Radioactivity	Recombinant	NA
NVS_NR_cAR	Chimp AR 3H-Methyltrienolone	chimp	367	AR		Radioactivity	Cell line	NA
OT_ERa_EREGFP_0120	OT_ERa_EREGFP_0120	human	2099	ESR1, ERE	Transactivation Or Cell Based	Protein-fragment Complementation Assay +/- 5% S9 activation	Cell line	HeLa
OT_ERa_EREGFP_0480	OT_ERa_EREGFP_0480	human	2099	ESR1, ERE			Cell line	HeLa
OT_ER_ERaERa_0480	OT_ER_ERaERa_0480	human	2099	ESR1			Cell line	HEK293T
OT_ER_ERaERa_1440	OT_ER_ERaERa_1440	human	2099	ESR1			Cell line	HEK293T
OT_ER_ERaERb_0480	OT_ER_ERaERb_0480	human	2099	ESR1, ESR2			Cell line	HEK293T
OT_ER_ERaERb_1440	OT_ER_ERaERb_1440	human	2099	ESR1, ESR2			Cell line	HEK293T
OT_ER_ERbERb_04	OT_ER_ERbERb_0	human	2099	ESR2			Cell line	HEK293T

80	480							
OT_ER_ERbERb_1440	OT_ER_ERbERb_1440	human	2099	ESR2			Cell line	HEK293T
OT_ERa_ERELUC_AG_1440	OT_ERa_ERELUC_AG_1440	human	2099	ESR1, ERE			Cell line	HeLa
ATG_ERE_CIS	Factorial reporter gene assay	human	2099	ESR1	In vitro (Cellular)	Reporter gene assay	Cell line	HepG2
ATG_ERa_TRANS	Factorial reporter gene assay	human	2099	ESR1	In vitro (Cellular)	Reporter gene assay	Cell line	HepG2
Tox21_ERa_LUC_BG1_Agonist	Tox21_ERa_LUC_BG1_Agonist	human	2099	ESR1	In vitro (Cellular)	Reporter gene assay	Cell line	BG1
Tox21_ERa_LUC_BG1_Antagonist	Tox21_ERa_LUC_BG1_Antagonist	human	2099	ESR1	In vitro (Cellular)	Reporter gene assay	Cell line	BG1
Tox21_ERa_BLA_Agonist_ratio	GAL4 BLAM Reporter gene assay: ERa	human	2099	ESR1	In vitro (Cellular)	Reporter gene assay	Cell line	HEK293H
Tox21_ERa_BLA_Antagonist_ratio	GAL4 BLAM Reporter gene assay: ERa	human	2099	ESR1	In vitro (Cellular)	Reporter gene assay	Cell line	HEK293H
Tox21_ERa_BLA_Agonist_ch1	Tox21_ERa_BLA_Agonist_ch1	human	NA	NA	In vitro (Cellular)	Reporter gene assay	Cell line	BLA
Tox21_ERa_BLA_Agonist_ch2	Tox21_ERa_BLA_Agonist_ch2	human	NA	NA	In vitro (Cellular)	Reporter gene assay	Cell line	BLA
ATG_ERRa_TRANS	Factorial reporter gene assay	human	2101	ESRRA	In vitro (Cellular)	Reporter gene assay	Cell line	HepG2
ATG_ERRg_TRANS	Factorial reporter gene assay	human	2104	ESRRG	In vitro (Cellular)	Reporter gene assay	Cell line	HepG2
ACEA_T47D_80hr_Positive	Real-Time Growth Kinetics in T47D cells	human	2099	ESR1	In vitro (Cellular)	RT-CES	NA	NA
OT_AR_ARELUC_AG_1440	OT_AR_ARELUC_AG_1440	human	367	AR, SRC-1	Cell Based	Protein-fragment Complementation Assay	Cell line	HEK293T

OT_AR_ARSRC1_0480	OT_AR_ARSRC1_0480	human	367	AR, SRC-1	Cell Based	Protein-fragment Complementation Assay	Cell line	HEK293T
OT_AR_ARSRC1_0960	OT_AR_ARSRC1_0960	human	367	AR, SRC-1	Cell Based	Protein-fragment Complementation Assay	Cell line	HEK293T
Tox21_AR_BLA_Agonist_ch1	Tox21_AR_BLA_Agonist_ch1	human	NA	NA	In vitro (Cellular)	Reporter gene assay	Cell line	BLA
Tox21_AR_BLA_Agonist_ch2	Tox21_AR_BLA_Agonist_ch2	human	NA	NA	In vitro (Cellular)	Reporter gene assay	Cell line	BLA
Tox21_AR_BLA_Agonist_ratio	Tox21_AR_BLA_Agonist_ratio	human	NA	AR	In vitro (Cellular)	Reporter gene assay	Cell line	HEK293H
Tox21_AR_BLA_Antagonist_ratio	Tox21_AR_BLA_Antagonist_ratio	human	NA	AR	In vitro (Cellular)	Reporter gene assay	Cell line	HEK293H
Tox21_AR_LUC_MDAKB2_Agonist	Tox21_AR_LUC_MDAKB2_Agonist	human	NA	NA	In vitro (Cellular)	Reporter gene assay	Cell line	MDAKB2
Tox21_AR_LUC_MDAKB2_Antagonist	Tox21_AR_LUC_MDAKB2_Antagonist	human	NA	NA	In vitro (Cellular)	Reporter gene assay	Cell line	MDAKB2
ATG_AR_TRANS	Factorial reporter gene assay	human	367	AR	In vitro (Cellular)	Reporter gene assay	Cell line	HepG2

Table S1. Details for the ToxCast ER and AR binding and transactivation assays selected during 3D-QSAR based prediction study

Excel File, Table S2a. The total chemical lists, CAS numbers, SMILES codes, corresponding ToxCast assay values, potency bins, and calculated RBA values (see Supplemental Code and Data Zip File for this table).

Table S2b. Summary performance of QSAR model predictions for **all** ToxCast II compounds against individual mammalian *in vitro* assays for Estrogen Receptor (ER) binding model v.03 (top) and Androgen Receptor (AR) binding model v.03 (bottom).

Estrogen Receptor (ER)	Human			Bovine			Mouse		
	Positive	Negative	Total	Positive	Negative	Total	Positive	Negative	Total
Positive	54	70	124	36	29	65	39	65	104
Negative	77	1644	1721	95	1685	1780	92	1649	1741
Total	131	1714	1845	131	1714	1845	131	1714	1845
Sensitivity (%)	54/124 = 43.6			36/65 = 55.4			39/104 = 37.5		
Specificity (%)	1644/1721 = 95.5			1685/1780 = 94.7			1649/1741 = 94.7		
Concordance (%)	(54+1644)/1845 = 92.0			(36+1685)/1845 = 93.3			(39+1649)/1845 = 91.5		
Androgen Receptor (AR)	Human			Chimp			Rat		
	Positive	Negative	Total	Positive	Negative	Total	Positive	Negative	Total
Positive	50	71	121	36	62	98	35	72	107
Negative	154	1483	1637	100	760	860	169	1482	1651
Total	204	1554	1758	136	822	958	204	1554	1758
Sensitivity (%)	50/121 = 41.3			36/98 = 36.7			35/107 = 32.7		
Specificity (%)	1483/1637 = 83.9			760/860 = 88.4			1482/1651 = 89.7		
Concordance (%)	(50+1483)/1758 = 95.5			(36+760)/958 = 83.0			(35+1482)/1758 = 86.3		

Table S3.

Estrogen Receptor (ER)	*Uterotrophic assay – All Compounds*			*Uterotrophic assay – In-domain Compounds*		
QSAR Model	Active	Inactive	Total	Active	Inactive	Total
Active	24	6	30	23	6	29
Inactive	5	7	12	2	5	7
Total	29	13	42	25	11	36
Sensitivity (%)	24/30 = 80.0			23/29 = 79.3		
Specificity (%)	7/12 = 58.3			5/7 = 71.4		
Concordance (%)	(24+7)/42 = 73.8			(23+5)/36 = 77.8		

Table S3a. Summary performance of QSAR model predictions for all 42 and 36 in-domain compounds with uterotrophic bioactivity.

Table S3b.

CASRN	Compound Name	Observed Value	Predicted Result	Total Domain	NVS_NR_bER	NVS_NR_hER	NVS_NR_mERa	Uterotrophic
57-63-6	17alpha-Ethinylestradiol	RBA>10%	Active	In domain	0.000245	5.41E-05	0.00185	Active
131-55-5	2,2',4,4'-Tetrahydroxybenzophenone	0.001<RBA<0.1%	Active	In domain	0.268	0.0534	0.176	Active
57-91-0	17alpha-Estradiol	RBA>10%	Active	In domain	0.000493	5.95E-05	0.0229	Active
50-28-2	17beta-Estradiol	RBA>10%	Active	In domain	0.000174	0.0229	0.00164	Active
80-05-7	Bisphenol A	0.1<RBA<10%	Active	In domain	0.389	0.131	0.15	Active
1478-61-1	Bisphenol AF	0.1<RBA<10%	Active	In domain	0.096	0.0449	0.0242	Active
77-40-7	Bisphenol B	0.1<RBA<10%	Active	In domain	0.149	0.0291	0.022	Active
56-53-1	Diethylstilbestrol	RBA>10%	Active	In domain	0.0229	0.0229	0.00632	Active
50-27-1	Estriol	RBA>10%	Active	In domain	0.00763	0.0229	0.0421	Active
53-16-7	Estrone	RBA>10%	Active	In domain	0.104	0.000795	0.00763	Active
446-72-0	Genistein	0.1<RBA<10%	Active	In domain	0.0983	0.0167	0.0901	Active

Table S3b. Eleven compounds that have ER binding at AC_{50} < 1 μM for all the three mammalian nuclear receptor binding assays were also active in the uterotrophic assay. They also belong to the training set data used to derive the ER QSAR model. The *in silico* prediction results including the total domain information as well as *in vitro* assay data are given.

Table S3c.

CASRN	Compound Name	Observed Value	Predicted Result	Total Domain	NVS_NR_ bER	NVS_NR_ hER	NVS_NR_ mERa	Uterotrophic
84-66-2	Diethyl phthalate	0<RBA<0.001%	Active	In domain	1000000	1000000	1000000	Inactive
84-75-3	Dihexyl phthalate	0<RBA<0.001%	Active	In domain	1000000	1000000	1000000	Inactive
84-61-7	Dicyclohexyl phthalate	0.001<RBA<0.1%	Active	In domain	1000000	1000000	1000000	Inactive
520-18-3	Kaempferol	0.001<RBA<0.1%	Active	In domain	1000000	0.214	1000000	Inactive

Table S3c. Four compounds (3 Phthalates and 1 Kaempferol) belonging to the training set were considered active in the ER *in vitro* assay used to derive the model but where inactive in uterotrophic bioactivity.

Table S4

Compound name	ER_Observed value	ER_Predicted result	ER_Total Domain	ER Alert group
1,2-Benzenedicarboxylic acid, di-C9-11-branched alkyl esters, C10-rich	0<RBA<0.001%	Not Active	In domain	Phthalates
4,4'-Sulfonyldiphenol	0.001<RBA<0.1%	Not Active	Out of Domain	AC,AD,Two nucleophilic sites
4-Dodecylphenol	0.1<RBA<10%	Not Active	Out of Domain	Alkylphenols
2,4-Di-tert-butylphenol	0.001<RBA<0.1%	Not Active	In domain	
4-Methylaniline	0<RBA<0.001%	Not Active	Out of Domain	Alkylamines
Benz(a)anthracene	0.001<RBA<0.1%	Not Active	Out of Domain	
Di(isononyl) phthalate branched	0<RBA<0.001%	Not Active	In domain	Phthalates
Diisobutyl phthalate	0<RBA<0.001%	Not Active	In domain	Phthalates
Dicofol	0.001<RBA<0.1%	Not Active	Out of Domain	
Methylparaben	0<RBA<0.001%	Not Active	In domain	Alkyl hydroxy benzoates
Kepone	0.001<RBA<0.1%	Not Active	Out of Domain	
Mono(2-ethylhexyl) phthalate	0.001<RBA<0.1%	Not Active	In domain	
Methoxychlor	0.001<RBA<0.1%	Not Active	In domain	
p-Cresol	0<RBA<0.001%	Not Active	Out of Domain	Alkylphenols
Phenol	0<RBA<0.001%	Not Active	Out of Domain	Alkylphenols
Propyl gallate	0<RBA<0.001%	Not Active	Out of Domain	AC,AD,Two nucleophilic sites ,Alkyl hydroxy benzoates

Table S4a. Sixteen compounds that were active experimentally and belonged to the training set but were predicted not active for ER binding.

Table S4b

**Compound name**	**ER_Observed value**	**ER_Predicted result**	**ER_Total Domain**	**ER Alert group**
1,2-Diphenylethanone	Not active	Active	In domain	Benzophenones
3-Ethylphenol	Not active	Active	Out of Domain	Alkylphenols
4,4'-Methylenebis(2,6-di-t-butylphenol)	Not active	Can't predict	In domain	#N/A
4-Octylphenol	Not active	Active	In domain	A_only_type
17-Methyltestosterone	Not active	Active	Out of Domain	Two nucleophilic sites
1-Hydroxypyrene	Not active	Active	Out of Domain	A_only Type
1-Naphthol	Not active	Active	Out of Domain	Alkylphenols
4-Chloro-3,5-dimethylphenol	Not active	Active	Out of Domain	A_only Type
4-Pentylaniline	Not active	Active	In domain	Alkylamines
2,2',6,6'-Tetrachlorobisphenol A	Not active	Active	Out of Domain	AC
Di(2-ethylhexyl) phthalate	Not active	Active	In domain	Phtalathes
Diallyl phthalate	Not active	Active	In domain	Phthalates
Dibutyl phthalate	Not active	Active	In domain	Phtalathes
Phenol red	Not active	Active	Out of Domain	AC
Phenolphthalin	Not active	Active	Out of Domain	AC

Table S4b. Fifteen Compounds that were not active experimentally and belonged to the training set but were predicted active for ER binding.

Table S5

Compound name	AR_Observed value	AR_Predicted result	AR_Total Domain	AR Alert group
4-Cumylphenol	0.001<RBA<0.1	Not Active	Out of Domain	
4-Dodecylphenol	0.001<RBA<0.1	Not Active	In domain	Alkylphenols
4-Phenylphenol	0.001<RBA<0.1	Not Active	Out of Domain	
Dibutyl hexanedioate	0.001<RBA<0.1	Not Active	In domain	(Prescreen)
Diisobutyl adipate	0.001<RBA<0.1	Not Active	In domain	(Prescreen)
Isoeugenol	0.001<RBA<0.1	Not Active	Out of Domain	
Metolachlor	0.001<RBA<0.1	Not Active	Out of Domain	
Kepone	0.001<RBA<0.1	Not Active	Out of Domain	
Methyl parathion	0.001<RBA<0.1	Not Active	Out of Domain	
Parathion	0.001<RBA<0.1	Not Active	Out of Domain	
Polyoxyethylene(10)nonylphenyl ether	0.001<RBA<0.1	Not Active	Out of Domain	
Triphenyl phosphate	0.001<RBA<0.1	Not Active	Out of Domain	

Table S5a. Twelve compounds that were active experimentally and belonged to the training set but were predicted not active for AR binding.

Compound name	AR_Observed value	AR_Predicted result	AR_Total Domain	AR Alert group
4-Ethylphenol	Not Active	Active	Out of Domain	Alkylphenols
Phenolphthalin	Not Active	Can't predict	Out of Domain	Two nucleophilic sites

Table S5b. Two compounds that were not active experimentally and belonged to the training set but were predicted active for AR binding.
